# A biodegradable PVA coating constructed on the surface of the implant for preventing bacterial colonization and biofilm formation

**DOI:** 10.1186/s13018-024-04662-7

**Published:** 2024-03-08

**Authors:** Zhonghua Lei, Haifeng Liang, Wei Sun, Yan Chen, Zhi Huang, Bo Yu

**Affiliations:** 1 Orthopedic and Traumatology Department, Zhujiang Hospital, Southern Medical University, Guangzhou, 510282 China; 2 Department of Orthopedics, The Sixth Peoples Hospital of Huizhou, Huizhou, 516211 China; 3 Department of Orthopedics, The Third Affiliated Hospital of Guangzhou Medical University, Guangzhou, 510150 China; 4 Ultrasound Medical Center, Zhujiang Hospital, Southern Medical University, Guangzhou, 510282 China; 5 Institute of Biomedical Engineering, School of Basic Medical Sciences, Central South University, Changsha, 410083 China

**Keywords:** PVA, Biofilm, Coating, Antibacterial, Implant

## Abstract

**Background:**

Bone implant infections pose a critical challenge in orthopedic surgery, often leading to implant failure. The potential of implant coatings to deter infections by hindering biofilm formation is promising. However, a shortage of cost-effective, efficient, and clinically suitable coatings persists. Polyvinyl alcohol (PVA), a prevalent biomaterial, possesses inherent hydrophilicity, offering potential antibacterial properties.

**Methods:**

This study investigates the PVA solution's capacity to shield implants from bacterial adhesion, suppress bacterial proliferation, and thwart biofilm development. PVA solutions at concentrations of 5%, 10%, 15%, and 20% were prepared. In vitro assessments evaluated PVA's ability to impede bacterial growth and biofilm formation. The interaction between PVA and mCherry-labeled *Escherichia coli* (*E. coli*) was scrutinized, along with PVA’s therapeutic effects in a rat osteomyelitis model.

**Results:**

The PVA solution effectively restrained bacterial proliferation and biofilm formation on titanium implants. PVA solution had no substantial impact on the activity or osteogenic potential of MC3T3-E1 cells. Post-operatively, the PVA solution markedly reduced the number of *Staphylococcus aureus* and *E. coli* colonies surrounding the implant. Imaging and histological scores exhibited significant improvements 2 weeks post-operation. Additionally, no abnormalities were detected in the internal organs of PVA-treated rats.

**Conclusions:**

PVA solution emerges as an economical, uncomplicated, and effective coating material for inhibiting bacterial replication and biofilm formation on implant surfaces, even in high-contamination surgical environments.

**Supplementary Information:**

The online version contains supplementary material available at 10.1186/s13018-024-04662-7.

## Introduction

The utilization of bone implants, encompassing internal fixation devices and artificial joints, stands as a pivotal cornerstone in the field of orthopedics, serving to stabilize body posture and restore the normal functionality of bones [1, 2]. Regrettably, the widespread deployment of these implants has brought about an unwelcome surge in implant-related infections, a complication that carries dire consequences, including impaired limb function, the necessity for multiple surgical interventions, and in severe cases, limb amputation [3].

Confronting implant infections within clinical practice presents formidable challenges. Conventional treatment approaches, such as antibiotic administration, debridement, revision surgeries, and, in the direst circumstances, amputation, have inherent shortcomings. These encompass extended hospitalization periods, the need for multiple surgical interventions, substantial financial burdens on patients and society, and soaring treatment costs [4–8]. In light of the profound impact of implant infections, it becomes increasingly clear that the prevention of such infections assumes greater significance than treatment methodologies. Hence, it becomes imperative to seek effective preventative measures in clinical practice.

Presently, clinical strategies for averting implant infections involve the optimization of preoperative risk factors, including the management of blood glucose levels, weight control, and the cessation of smoking and alcohol consumption, among others. Furthermore, preoperative skin preparation, perioperative antibiotic prophylaxis, the maintenance of a sterile surgical environment, and strategies aimed at preventing prosthesis-related infections are frequently employed [9–11]. Although antibiotic-coated implants are commonly utilized for infection prevention, their efficacy is limited by a restricted antibiotic release profile and a narrow spectrum of antibacterial activity, ultimately contributing to the emergence of antibiotic resistance [12].

Recent research endeavors have concentrated on the development of modified implants with intrinsic infection-prevention capabilities [13]. These innovations encompass the integration of antibiotics, antimicrobial peptides, silver ion coatings [14–17], and intelligent antibacterial coatings induced by physical mechanisms such as photocatalysis and ultrasonic catalysis [18, 19]. However, these methods are not without their limitations, which include drug resistance, a limited range of antibacterial activity, potential biotoxicity, and intricate manufacturing processes, all of which impede their widespread clinical application [20–22].

One of the causes of infection of bone implants may be the natural attachment of bacteria during the operation [23, 24]. Subsequently, the bacteria further combine with the implant through microbial surface components recognizing adhesive matrix molecules (MSCRAMMs) and form a biofilm [25]. Immune evasion and antibiotic resistance caused by biofilm formation are also the main reasons why implant infection is difficult to treat [25, 26]. Fascinatingly, the concept of aiding host cells in winning the “race for the surface” presents a promising avenue for preventing implant infections. Coined by Anthony G. Gristina, the term “race for the surface” illustrates the competition between host cells and microorganisms for dominance on the implant surface [27]. Once the surface is occupied by host cells, it becomes a formidable challenge for bacteria to establish colonization. However, this competition tends to be skewed in favor of bacteria during the surgical implantation process, granting them an opportunity to gain the upper hand [28].

Aligned with the “race for the surface” concept, the present study embarks on a mission to craft an absorbable solution for medical device implants capable of thwarting bacterial colonization during surgery. Our innovative approach entails the creation of a coating that, upon dissolution, eliminates bacteria adhering to it, thereby averting bacterial colonization and biofilm formation on the implant's inner surface. To realize this vision, we have selected polyvinyl alcohol (PVA) molecules with a molecular weight of 27,000 as the foundational element for our antibacterial adhesion solution. PVA, an FDA-approved synthetic macromolecule for human clinical use [29], boasts a well-established track record in diverse bioengineering applications, including artificial tears, tissue adhesion barriers, hemodialysis, and bone implants, owing to its favorable attributes, such as low biotoxicity, high hydrophilicity, and renal filtration compatibility [30–38]. Furthermore, PVA has proven efficacy as a constituent in antibacterial composites [39–41].

**Scheme 1.**
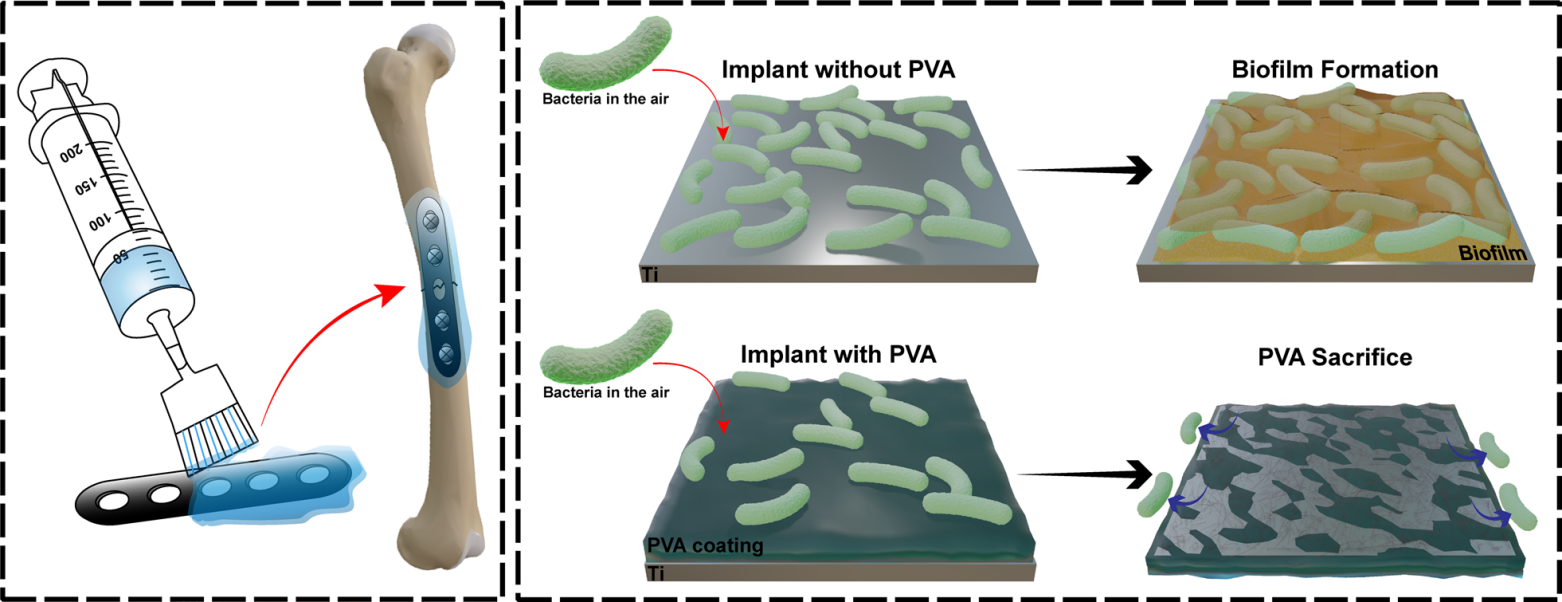
The scheme shows the experimental design of this study

In this study, we conducted a systematic investigation of PVA solutions at different concentrations, both in vitro and in vivo, to assess their bacteriostatic and bactericidal properties as well as their biocompatibility. This included the preparation of PVA solutions at various concentrations, followed by the evaluation of their dissolution rates under in vitro conditions. Subsequently, we examined the effectiveness of the PVA solution coating on titanium plates in inhibiting biofilm formation. Our investigation culminated in a comprehensive assessment of the protective capacity of the PVA solution using a rat model of contaminated implants (Scheme 1).

## Materials and methods

### Preparation of PVA solution

Polyvinyl alcohol (PVA, sigma, USA) aqueous solutions were heated to 90 °C and dissolved at concentrations of 5 wt%, 10 wt%, 15 wt%, and 20 wt%. Additionally, the osmotic pressure of each solution was adjusted to a range of 286–296 mmol/L by adding sodium chloride, sodium hydroxide, and hydrochloric acid, while maintaining a pH level between 7.2 and 7.4. Following these adjustments, the PVA solution underwent sterilization using an autoclave sterilizer.

### Assessment of PVA solution dissolution rate

To determine the dissolution rate of the PVA solution, we employed a weight change measurement methodology. Implants coated with the PVA solution were carefully submerged in phosphate-buffered saline (PBS) and positioned within a stable incubator set at 37 °C. Dissolution assessments were conducted at specified time intervals, including 0, 10, 20, 30, 60, and 120 min. After each designated period, the implants were gently extracted from the PBS solution and precisely weighed.

The weight loss rate of the PVA coating was quantified using the following formula:

1 $$Weight \;change\left( \% \right) = \frac{{\left( {Weight\left( {0 \min } \right) - Weight\left( {implant} \right)} \right) - \left( {Weight\left( {x \min } \right) - Weight\left( {implant} \right)} \right)}}{{Weight\left( {0 \min } \right) - Weight\left( {implant} \right)}} \times 100\%$$

### Visualization of mobility and wall adhesion behavior

To investigate the flow characteristics of the PVA solution, each prepared solution was placed in a sample bottle and allowed to settle until it achieved a state of non-flowing equilibrium. Subsequently, the sample bottle was positioned vertically, and photographic documentation was conducted to record the state and appearance of the PVA material.

### Rheological analysis of PVA solution

The rheological properties of the PVA solutions at a controlled temperature of 37 °C were assessed using a rheometer (Malvern, UK). Parallel plate geometry was adopted, with a diameter of 40 mm and a gap of 5 mm. Before testing, the specimens were held at this temperature for 15 min to eliminate their thermal and shear histories. Frequency sweep tests were carried out in an oscillatory mode over the range from 0.1 to 20 rad/s.

### Evaluation of PVA's antibacterial effects

To investigate the inhibitory effect of PVA on bacterial proliferation, the following experimental groups were established: 1. PBS group; 2. 5% PVA group; 3. 10% PVA group; 4. 15% PVA group; 5. 20% PVA group. Initially, 24-well plates were coated with PVA solutions of varying concentrations, followed by the addition of *Escherichia coli* (*E. coli*) or *Staphylococcus aureus* (*S. aureus*) suspensions at a concentration of 5 × 10^7^ CFU/ml. The plates were then incubated at 37 °C for 2 h before conducting plate spreading assays.

To further simulate the antibacterial effect of PVA solutions in practical applications, different concentrations of PVA solutions were evenly spread on the titanium plate through the syringe to form a PVA coating (see Additional file 1: Fig. S1). Subsequently, the titanium plates were fully immersed in suspensions of *Escherichia coli* or *Staphylococcus aureus* at a concentration of 5 × 10^7^ CFU/ml. Similar to the previous procedure, the plates were incubated at 37 °C for 2 h before conducting plate spreading assays.

According to the GB4789.2-94 standard, the mixture of each group was diluted 10 times after incubation for 2 h, and the dilution range was from 10^1^ to 10^10^. Using the plate spreading technique, each dilution was evenly spread onto agar plates, and after 24 h of further incubation, the bacterial colony counts on the agar plates were quantified.

### Assessment of PVA's potential to prevent bacterial biofilm formation

To investigate the potential of PVA solutions in preventing the formation of biofilms on the surfaces of plastic instruments, the following experimental groups were established: 1. PBS group; 2. 5% PVA group; 3. 10% PVA group; 4. 15% PVA group; 5. 20% PVA group. Similar to the previously described procedure, PVA solutions were initially coated at the bottom of well plates, followed by the addition of suspensions of *Escherichia coli* or *Staphylococcus aureus* at a concentration of 5 × 10^7^ CFU/ml into the wells. All samples were then incubated at 37 °C for 2 h. Subsequently, the PVA solution and bacterial suspensions were aspirated, and the wells or titanium plates were washed three times with PBS to remove planktonic bacteria. The wells were then filled with, or the titanium plates were immersed in a 0.25% crystal violet solution, and samples were stained for 20 min at room temperature. Afterward, samples were washed three times with PBS to remove excess crystal violet. Finally, samples were dried in a 37 °C oven for 1 h.

To simulate the ability of PVA solutions to prevent bacterial biofilm formation on the surface of implanted titanium plates, we chose titanium plates with screw-fixed holes. The experimental procedure involved initially coating the area surrounding the screw-fixed holes on the titanium plates with PVA solutions to form a coating. Then, the titanium plates with screw-fixed holes were immersed in bacterial suspensions. Similar to the previous procedure, all samples were incubated at 37 °C for 2 h, and subsequently, the PVA solution and bacterial suspensions were aspirated, and the plates were washed three times with PBS to remove planktonic bacteria. The titanium plates with screw-fixed holes were then immersed in a 0.25% crystal violet solution and stained for 20 min at room temperature. Afterward, samples were washed three times with PBS to remove excess crystal violet. Finally, samples were dried in a 37 °C oven for 1 h.

To quantify biofilm formation, an appropriate volume of anhydrous ethanol was added to each sample to ensure complete dissolution of crystal violet. The optical density 600 nm (OD600) of the crystal violet-ethanol solution was measured in a microplate reader (BioTek, USA) as a quantitative indicator of biofilm formation.

### Fluorescent labeling and visualization of bacterial interactions

To investigate the interaction between PVA and bacteria, we employed fluorescently labeled bacteria to monitor changes in their behavior in the presence of PVA.

#### Confocal observation of biofilms

*Escherichia coli*, genetically engineered to express mCherry protein (referred to as *E. coli*-mCherry) at a concentration of 5 × 10^7^ CFU/ml, was prepared. Following the previously described procedure, PVA-coated and uncoated titanium plates were immersed in the *E. coli*-mCherry suspension for 2 h. Subsequently, the plates underwent three washes with PBS to remove any unattached bacteria. Finally, the distribution of *E. coli*-mCherry on the titanium plates was visualized using a laser confocal microscope (Nikon, Japan).

#### Fluorescence detection of *E. coli*

*Escherichia coli*-mCherry suspension (5 × 10^7^ CFU/ml) was mixed with PBS and various concentrations of PVA solution in equal proportions. After incubation at 37 °C for 24 h, the fluorescence intensity of each sample was measured using a fluorescence luminescence instrument (Alliance Q9 Chroma, England). Gray value analysis was performed using Image J software. Additionally, titanium plates coated with a 20% PVA solution and uncoated titanium plates were immersed in *E. coli*-mCherry suspension with a concentration of 5 × 10^7^ CFU/ml for 2 h. The plates were then subjected directly to fluorescence detection using a fluorescence luminescence instrument (Alliance Q9 Chroma, England).

#### Confocal observation of *E. coli* distribution within PVA

To make PVA fluorescent, we replaced PVA with fluorophore pyrene formaldehyde modified PVA (modified 1%) and equipped with 20% pyrene formaldehyde modified PVA solution. Subsequently, the *E. coli*-mCherry solution (5 × 10^7^ CFU/ml) was gently placed onto the 20% pyrene formaldehyde modified PVA solution. The distribution of live and dead bacteria was observed using a fluorescence microscope. The pyrene formaldehyde-labeled PVA and *E. coli*-mCherry were visualized using a laser confocal microscope (Nikon, Japan).

### Cytotoxicity assessment

To assess the cytotoxicity of PVA, primary chondrocytes, RAW264.7, and MC3T3-E1 cells were co-cultured with PVA within a Transwell chamber for 24 h. Chondrocytes were cultured in Dulbecco’s modified Eagle medium/F12 (DMEM/F12, Gibco, USA) containing 1% penicillin streptomycin (P/S, Gibco, USA) and 10% Fetal Bovine Serum (FBS, Gibco, USA). RAW264.7 were cultured in DMEM (Gibco, USA) containing 1% P/S and 10% FBS. MC3T3-E1 were cultured in Minimum essential medium-α (MEM-α, Gibco, USA) containing 1% P/S and 10% FBS. All cells were cultured at 37 °C and 5% CO_2_. The cytotoxicity of the PVA solution was evaluated using the Cell Counting Kit-8 (CCK-8) method.

Following the co-culture period, the cells were detached using trypsin, centrifuged, and subsequently reseeded into a 96-well plate at a density of 5 × 10^3^ cells/well. The CCK-8 working solution was prepared by combining the CCK-8 reagent with the appropriate culture medium (DMEM/F12 containing 1% P/S and 10% FBS for chondrocytes; DMEM containing 1% P/S and 10% FBS for RAW264.7 and MEM-α containing 1% P/S and 10% FBS for MC3T3-E1) in a 1:10 ratio. Subsequently, the CCK-8 working solution was added to each well, and the cells were incubated under controlled conditions for 1 h. Finally, the optical density at 450 nm (OD450nm) for each sample was determined using a microplate reader (BioTek, USA).

### ALP activity measurement and ALP staining

Similarly, MC3T3-E1 cells were co-cultured with 5–20% PVA solutions using a Transwell chamber in MEM-α containing 1% P/S and 10% FBS for 24 h. Following this, all the samples were washed three times with PBS and cultured in an osteogenic induction medium [MEM-α containing 1% P/S, 10% FBS, 50 μg/ml l-ascorbic acid (Sigma, USA), 10 mM β-glycerophosphate (Sigma, USA) and 10nM dexamethasone (Sigma, USA)]. After 14 days of culture, ALP activity and ALP staining were performed.

For ALP activity detection, all cells were lysed using a 1% Triton X-100 solution. Three groups were set up, including a blank group (dd water), a detection group (cell sample), and a standard group (0.1 mg/ml phenol standard solution). The color reaction was initiated, and the optical density at 520 nm (OD520nm) of all groups was measured using an enzyme labeling instrument. A standard curve of protein concentration was established, and the protein concentration of all cell samples was determined using the BCA method. The ALP activity value of each group was calculated using the following formula:

2 $${\text{ALP}}\;activity\left( {{\text{IU/mg}}\; total\;protein} \right) = \frac{Test\;OD - Blank\;OD}{{Standard\;OD - Blank\;OD}} \times 0.1\,{\text{mg/ml}} \div Protein \;concentrarion\left( {\text{gprot/ml}} \right)$$

Simultaneously, parallel samples were fixed in a fixing solution for 3 min, according to the instructions of the reagent. The working solution was then added to cover all the samples, which were incubated at 37 °C away from light for 15 min. Finally, the samples were washed three times with PBS and observed under an optical microscope (Leica, USA).

### Alizarin red S staining

As described above, MC3T3-E1 were co-cultured with 5–20% PVA solutions using a Transwell chamber in MEM-α containing 1% P/S and 10% FBS for 24 h. Then MC2T2-E1 were cultured in osteogenic induction medium (MEM-α containing 1% P/S, 10% FBS, 50 μg/ml l-ascorbic acid, 10 mM β-glycerophosphate and 10 nM dexamethasone) for 21 days [42–44], and finally were stained with Alizarin Red S (Leagene Biotechnology, China). All samples were fixed with 95% ethanol for 10 min. After thorough air-drying, the samples were immersed in an Alizarin Red S dye solution and incubated at room temperature for 15 min. Subsequently, the samples were quickly washed with distilled water and air-dried again. Finally, the samples were observed under an optical microscope (Leica, USA).

### Rat model of osteomyelitis

All animal experiment procedures were conducted in compliance with the guidelines approved by the Institutional Animal Care and Use Committee (IACUC) of Zhujiang Hospital, Southern Medical University. Seventy-five male Sprague Dawley rats weighing 300 g were randomly divided into five groups: Control group, *S. aureus* group, *E. coli* group, PVA + *S. aureus* group, and PVA + *E. coli* group. The animals were housed in a controlled environment with a 12-h light–dark cycle. They had ad libitum access to food and water, and the bedding was changed every 3 days. Before use, the concentration of *S. aureus* and *E. coli* was adjusted to 5 × 10^7^ CFU/ml, and the implants were sterilized using high-pressure steam. The rats were anesthetized with pentobarbital sodium via intraperitoneal injection.

After achieving satisfactory anesthesia, the right lower limbs of the rats were prepared by skin disinfection and aseptic draping. A 2 cm incision was made in the anterolateral thigh to expose the subcutaneous fascia. The quadriceps femoris was bluntly separated to the middle and lower part of the femur. After tapping, pre-prepared screws and steel plates were inserted. Implants in the bacterial groups were contaminated with *E. coli* or *S. aureus* before implantation, while implants in the PVA + bacteria groups were wrapped with PVA using a syringe and then contaminated with *E. coli* or *S. aureus*. No antibiotics were administered postoperatively.

At 2 h, 24 h, and 2 weeks after the operation, animals in each group were euthanized by cervical dislocation. Five rats from each group were euthanized at each time point. After skin disinfection, the incisions were reopened, and the muscles were gently dissected with tweezers to expose the implants. A sterile swab was then used to gently rub the surface of the implant by rotating it clockwise three times and counterclockwise three times. The swab was subsequently immersed in sterile normal saline, sealed, and transferred to a biosafety cabinet for further analysis, including flat plate coating.

### Histological and imaging examination in vivo

Two weeks after the operation, the rats underwent sampling of bacteria around the implant. The lower part of the femur was dissected, and the surrounding muscles and ligaments were removed. The femur was then fixed using 4% paraformaldehyde. Subsequently, anterior and lateral X-ray scans of the femur were performed using a SCANCO system (Switzerland).

For histological examination, all samples were fixed, decalcified using a decalcification solution (Leagene Biotechnology, China), and embedded in paraffin. The paraffin-embedded samples were then sliced into sections. Hematoxylin and eosin (H&E) staining was performed on these sections to observe the extent of inflammatory cell infiltration in the bone marrow cavity.

### Data statistics

All data are expressed as mean ± standard deviation. The Student’s t-test was used to analyze the differences between the two groups. One-way ANOVA and Tukey’s test were used to compare among groups. A *p* value less than 0.05 was considered statistically significant. At least three independent samples were used for all in vitro experiments and at least five independent samples were used for in vivo experiments.

## Results

### Adhesion properties of PVA solutions

**Fig. 1:**
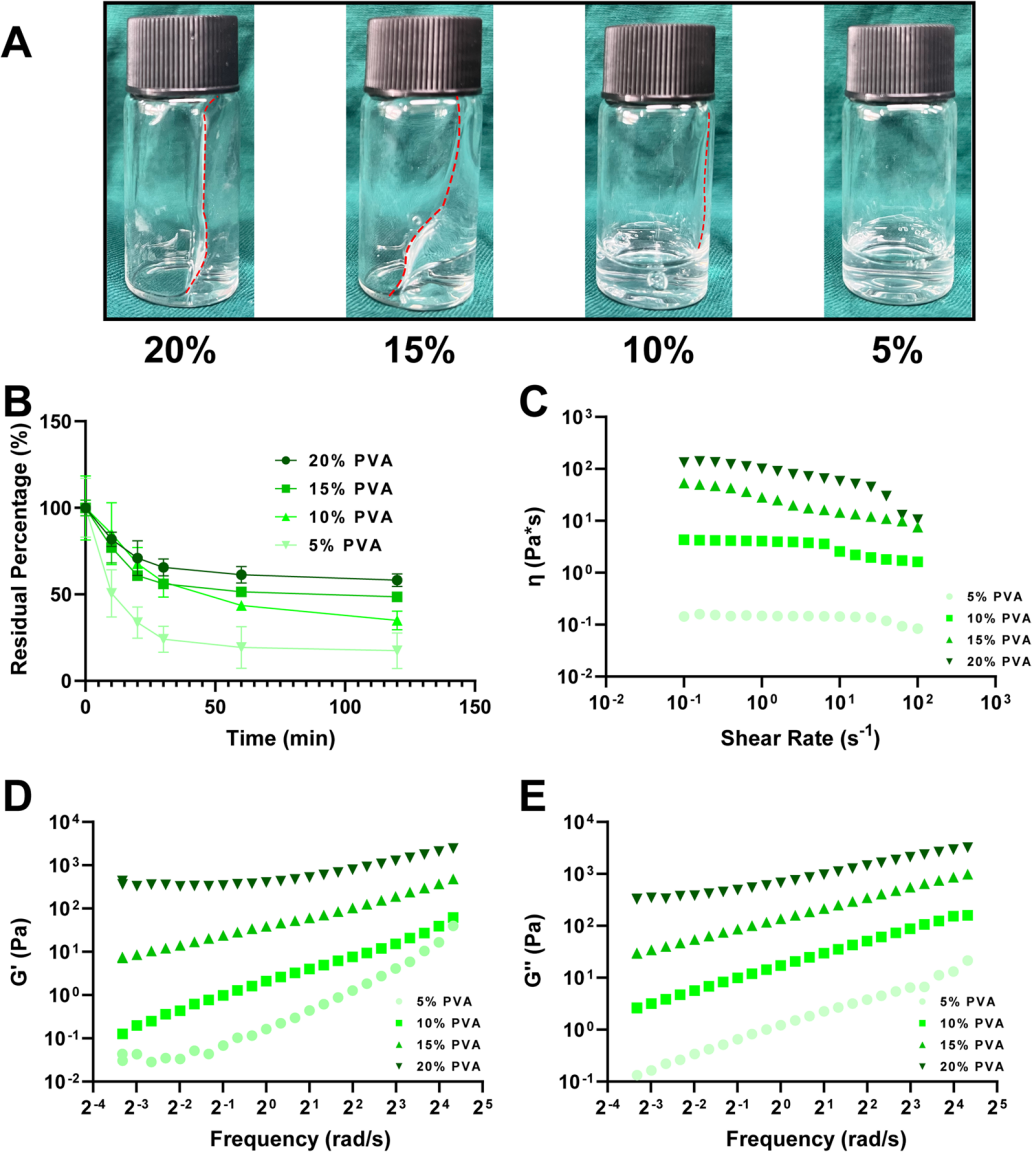
Characterization of PVA solution. **A** Adhesion of different concentrations of PVA solution to the bottle wall. **B** Dissolution rate of PVA coating on implants in PBS at 37 °C. **C** Shear viscosities curves of PVA solutions at 37 °C. Elastic (**D**) and viscous (**E**) component curves of shear modulus of different concentrations of PVA at 37 °C

Figure 1A illustrates the adhesion behavior of PVA solutions with varying concentrations to the walls of the sample bottle during position transformation. Our findings reveal that PVA solutions within the concentration range of 15–20 wt% exhibit prolonged adherence to vertical container surfaces, indicating excellent compatibility with instrumental surfaces.

### Dissolution rate of PVA solution

The results presented in Fig. 1B demonstrate a clear inverse relationship between the concentration of PVA and the dissolution rate of the PVA solution in PBS. Specifically, higher concentrations of PVA result in more viscous solutions, leading to slower rates of dilution. Conversely, lower concentrations of PVA solutions facilitate quicker dilution.

### Shear viscosity and shear modulus of PVA solution

Figure 1C showcases the shear viscosity of the PVA solution at a temperature of 37 °C. It is evident that higher concentrations of PVA correspond to increased shear viscosity at identical shear rates. Furthermore, Fig. 1D and E illustrate how both the elastic and viscous components of the shear modulus rise with escalating PVA concentrations at the same frequency.

### Antibacterial and antibiofilm effects of PVA on well plates

**Fig. 2:**
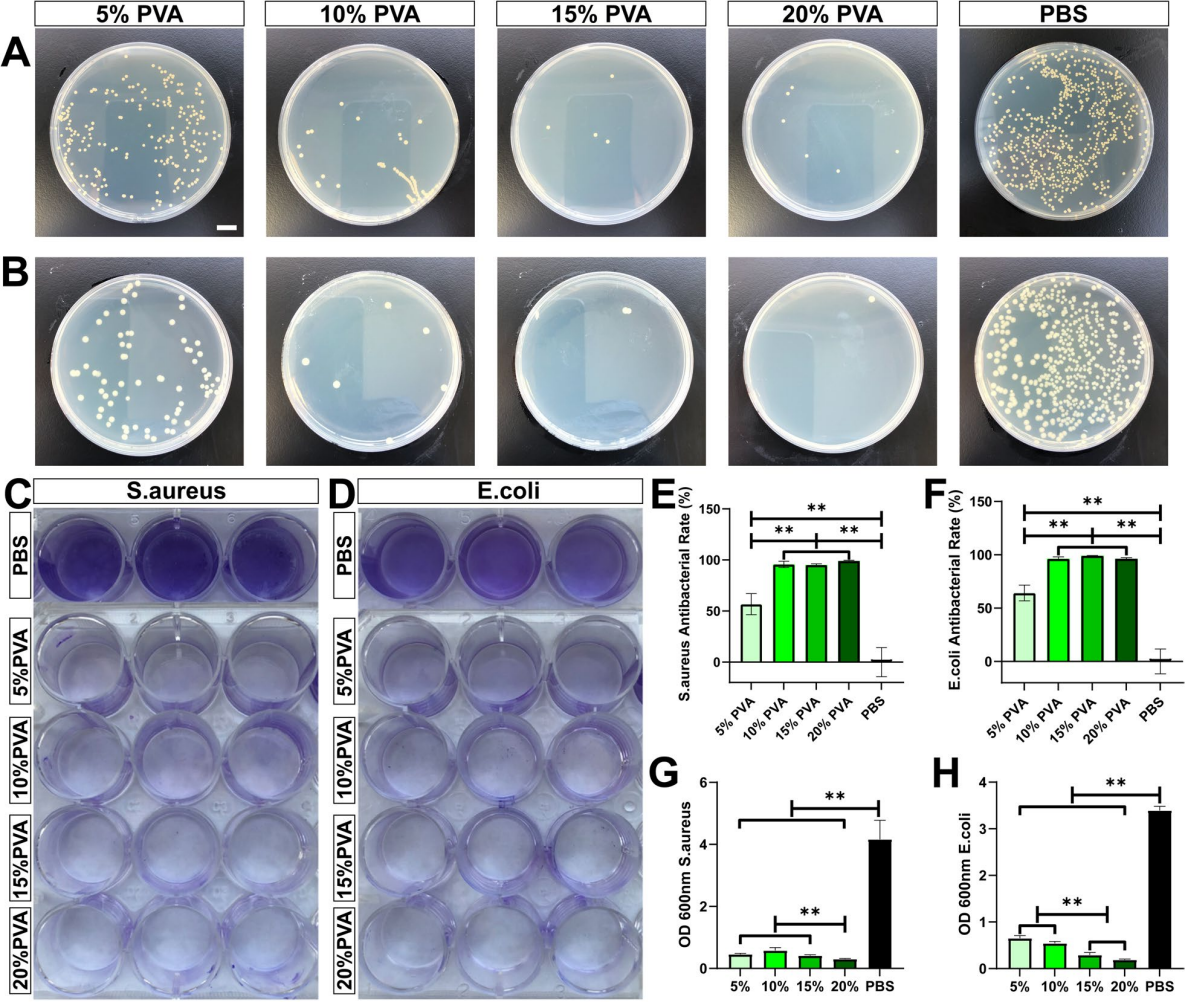
Antibacterial and Antibiofilm Effects of PVA Solution on 24-well Plate. **A** Plate coating results of *S. aureus* (bar = 10 mm) and **E** corresponding counting results. **B** Plate coating results of *E. coli* and **F** corresponding counting results. **C** Formation of *S. aureus* biofilm and **G** quantitative analysis. **D** Formation of *E. coli* biofilm and **H** quantitative analysis. Data are presented as means ± SD. Significant differences are indicated as **(*p* < 0.05)

To simulate the preventive effects of PVA solutions on bacterial colonization and biofilm formation on the surfaces of plastic products, we conducted experiments using cell culture plates. The presence of various concentrations of PVA solutions resulted in a significant reduction in the concentrations of both *S. aureus* and *E. coli* after 2 h of incubation. Notably, the bacteriostatic rate of 5%PVA is significantly lower than that of other concentrations of PVA (see Fig. 2A, B, E, and F). Importantly, PVA solutions ranging from 5 to 20% demonstrated a remarkable inhibitory effect on the formation of bacterial biofilms, whether it was *S. aureus* (see Fig. 2C) or *E. coli* (see Fig. 2D). It's worth highlighting that the inhibitory effect of 20% PVA on *S. aureus* biofilm formation significantly surpassed that of other PVA concentrations (see Fig. 2G). In the case of *E. coli* biofilm formation, the most effective concentrations were observed to be 15% and 20% PVA, with the inhibitory effect decreasing as the PVA concentration decreased (see Fig. 2H).

### Antibacterial and antibiofilm effects of PVA on titanium plates

**Fig. 3:**
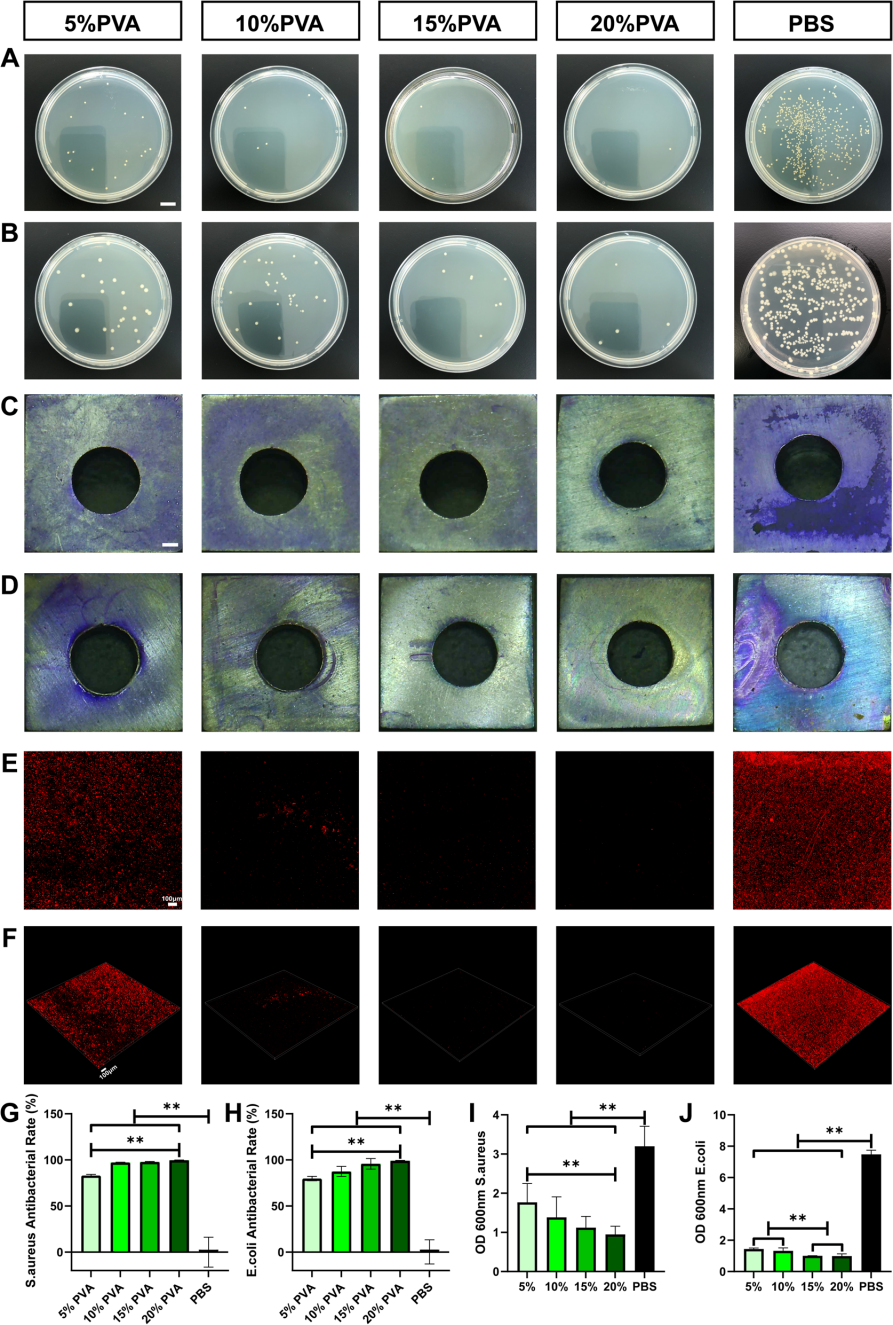
Antibacterial and Antibiofilm effects of PVA solution on Titanium Plate. **A** *S. aureus* plate coating results (bar = 10 mm) and corresponding counting results (**G**). **B** *E. coli* plate coating results and corresponding counting results (**H**). **C** Formation of *S. aureus* biofilm (bar = 1 mm) and quantitative analysis (**I**). **D** Formation of *E. coli* biofilm and quantitative analysis (**J**). **E** Multi-layer superposition map of *E. coli*-mCherry planted on titanium plate (bar = 100 μm). **F** 3D reconstruction map of *E. coli*-mCherry planted on titanium plate (bar = 100 μm). Data are represented as means ± SD. Significant differences are indicated as **(*p* < 0.05)

To assess the protective effect of PVA solution coating on metal implants, a titanium plate was evenly coated with PVA solution using a syringe to ensure complete coverage. After a 2-min deposition period, excess PVA solution was removed, and the titanium plate was exposed to bacterial contamination. The results revealed that each concentration of PVA solution significantly reduced the presence of bacteria on the titanium plate, with 20% PVA exhibiting the most pronounced antibacterial effect (see Fig. 3A, B, G, and H). Furthermore, the PVA solution on the titanium plate effectively inhibited the formation of bacterial biofilms (see Fig. 3C, D). Remarkably, higher concentrations of PVA displayed stronger anti-biofilm capabilities (see Fig. 3I, J).

To gain insights into the mechanism behind PVA's inhibition of bacterial growth and its anti-biofilm properties, we conducted further experiments using *E. coli* labeled with mCherry protein (referred to as *E. coli*-mCherry). Laser confocal microscopy was employed to visualize biofilm formation on the titanium plate surface. The results (see Fig. 3E, F) corroborated the findings obtained through crystal violet staining. In the control group, a substantial number of bacteria adhered to the titanium plate surface, forming a dense biofilm. In contrast, in the PVA-treated groups, the number of bacteria on the titanium plate surface decreased progressively with increasing PVA concentration.

In the 5% PVA group, although a considerable number of bacteria were still present on the titanium plate surface, the biofilm formed by the bacteria no longer covered the entire plate. In the 10% PVA group, a sheet-like biofilm was locally formed on the titanium plate surface. Notably, the 15% PVA and 20% PVA groups exhibited only sporadic red fluorescence, indicating a significant reduction in bacterial presence on the titanium plate surface. We attribute this phenomenon to the hydrogen bonds and van der Waals forces between molecules in high concentration PVA solutions, which form tiny and dense pore structures [45]. This makes it difficult for bacteria to penetrate the PVA coating, proliferate and form a biofilm on the surface of the titanium plate [46].

**Fig. 4:**
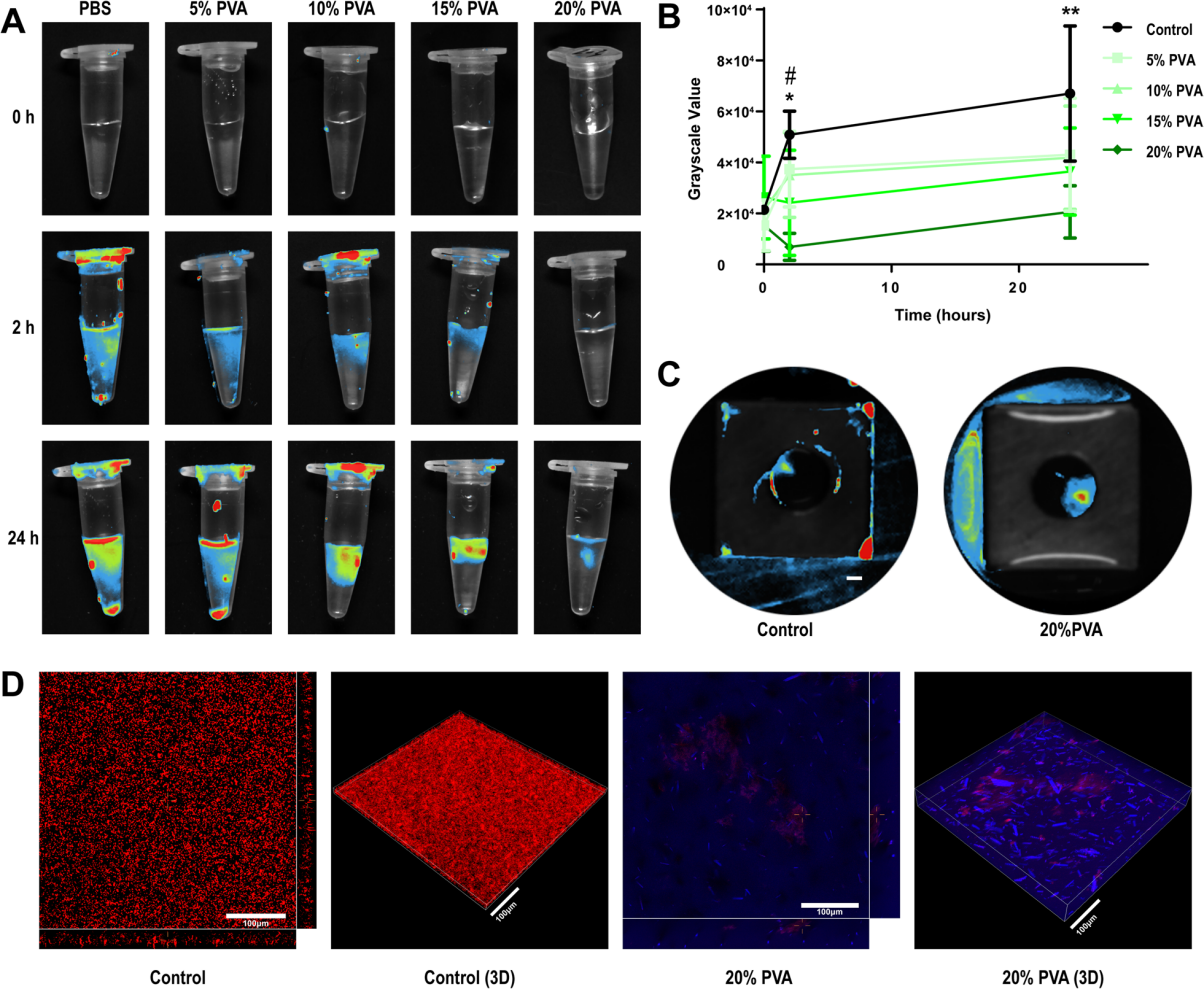
**A** Photoluminescence of *E. coli*-mCherry in different concentrations of PVA solution at different time points; **B** Quantitative results of grayscale values of each group in (**A**). **C** Photoluminescence diagram of titanium plate contaminated by *E. coli*-mCherry for 2 h (bar = 1 mm). **D** Confocal and 3D reconstruction map of *E. coli*-mCherry with PBS or 20%PVA labeled with pyrene formaldehyde. Bar = 100 μm. Data are represented as means ± SD. Significant differences between 15%PVA group and control group are indicated as ^#^(*p* < 0.05). Significant differences between 20%PVA group and control group, 5%PVA group and 10%PVA group are indicated as *(*p* < 0.05). Significant differences between 20%PVA group and control group are indicated as **(*p* < 0.05)

To investigate whether PVA can inhibit bacterial proliferation, we conducted experiments by mixing the bacterial suspension with different concentrations of PVA and monitoring bacterial growth over 24 h. As shown in Fig. 4A, the control group exhibited a gradual accumulation of bacteria at the bottom after 2 h of culture. In contrast, the presence of a 10% PVA solution inhibited bacterial growth, and the inhibitory effect became more pronounced with higher PVA concentrations. Semi-quantitative analysis (Fig. 4B) revealed a significant difference in the bacteriostatic effect between the 15% and 20% PVA groups compared to the other groups. After 24 h of culture, only the 20% PVA group showed a statistically significant difference compared to the control group.

To track the behavior of bacteria on the titanium plate, fluorescence imaging was performed on the control group and the 20% PVA group (Fig. 4C). The 20% PVA group exhibited PVA solution still adhering to the surface of the titanium plate, and bacteria were observed to be carried away from the implant with the flow of the PVA solution. This indicates that even when the titanium plate is immersed in a high concentration of bacterial liquid, it remains protected by the presence of 20% PVA, preventing bacterial colonization in a short time. In contrast, the control group showed localized areas of high bacterial accumulation on the surface of the titanium plate, indicating the formation of a biofilm.

To further investigate the spatial relationship between PVA and bacteria, we labeled PVA with pyrene formaldehyde through grafting. The bacterial suspension was then added to the 20% PVA-coated surface, and confocal scanning was performed layer by layer. The results demonstrated that 20% PVA not only significantly inhibited bacterial proliferation but also confined the bacteria to the top of the PVA coating (Fig. 4D).

### Impact of PVA on bone tissue-related cells in vitro

**Fig. 5:**
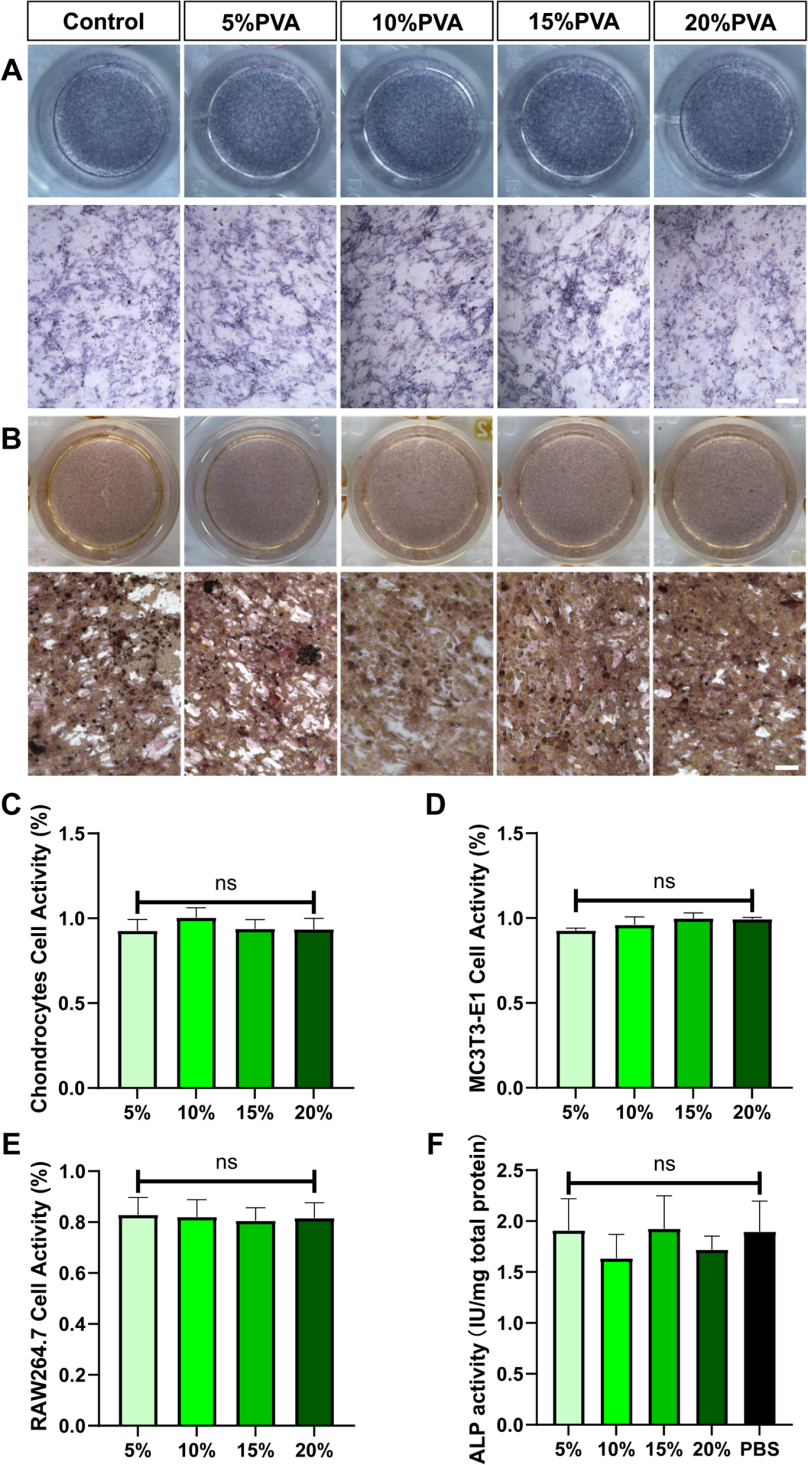
Effects of PVA Solution on Cell Survival and Osteogenic Differentiation in vitro. **A** ALP staining results. **B** Alizarin red S staining results; bar = 100 μm. **C** Survival rate of MC3T3-E1 cells under the influence of PVA solution. **D** Survival rate of chondrocytes under the influence of PVA solution. **E** Survival rate of RAW264.7 cells under the influence of PVA solution. **F** Results of ALP activity test. Data are represented as means ± SD. ns means no significance

To further assess the influence of PVA on musculoskeletal-related cells, we conducted co-cultures of osteoblasts, chondrocytes, and macrophages with PVA solutions within Transwell chambers. Encouragingly, the viability of MC3T3-E1 osteoblasts and chondrocytes remained notably high, with survival rates approaching 100% when exposed to PVA solutions ranging from 5 to 20% (see Fig. 5C, D), indicating the absence of significant cytotoxicity. However, the viability of RAW 264.7 macrophages displayed a decline when exposed to PVA solutions, particularly in the 15% and 20% PVA groups, where the cell survival rate dropped to below 80% (see Fig. 5E).

Analysis of alkaline phosphatase (ALP) staining indicated that the early osteogenic differentiation of MC3T3-E1 cells was not significantly affected by PVA solutions ranging from 5 to 20% (see Fig. 5A). However, ALP activity in the 10% PVA group (1.6341 IU/mg total protein; SD 0.235) and 20% PVA group (1.7201 IU/mg total protein; SD 0.132) exhibited a slight reduction compared to the PBS group (1.8963 IU/mg total protein; SD 0.301), although these differences did not reach statistical significance (see Fig. 5F). Furthermore, Alizarin red S staining demonstrated that PVA solutions did not interfere with the deposition of calcium nodules by MC3T3-E1 cells (see Fig. 5B).

The high biocompatibility and non-toxicity of PVA can explain the results. As a clinical biomaterial approved by FDA [29], the safety of PVA has been fully verified, so PVA does not inhibit the osteogenic differentiation of MC3T3-E1. On the other hand, PVA has not been found to have osteogenic activity, which is similar to the work of Hou et al. [47]. Therefore, PVA does not promote osteogenic differentiation of MC3T3-E1.

### Antibacterial and antibiofilm effects of PVA in vivo

**Fig. 6:**
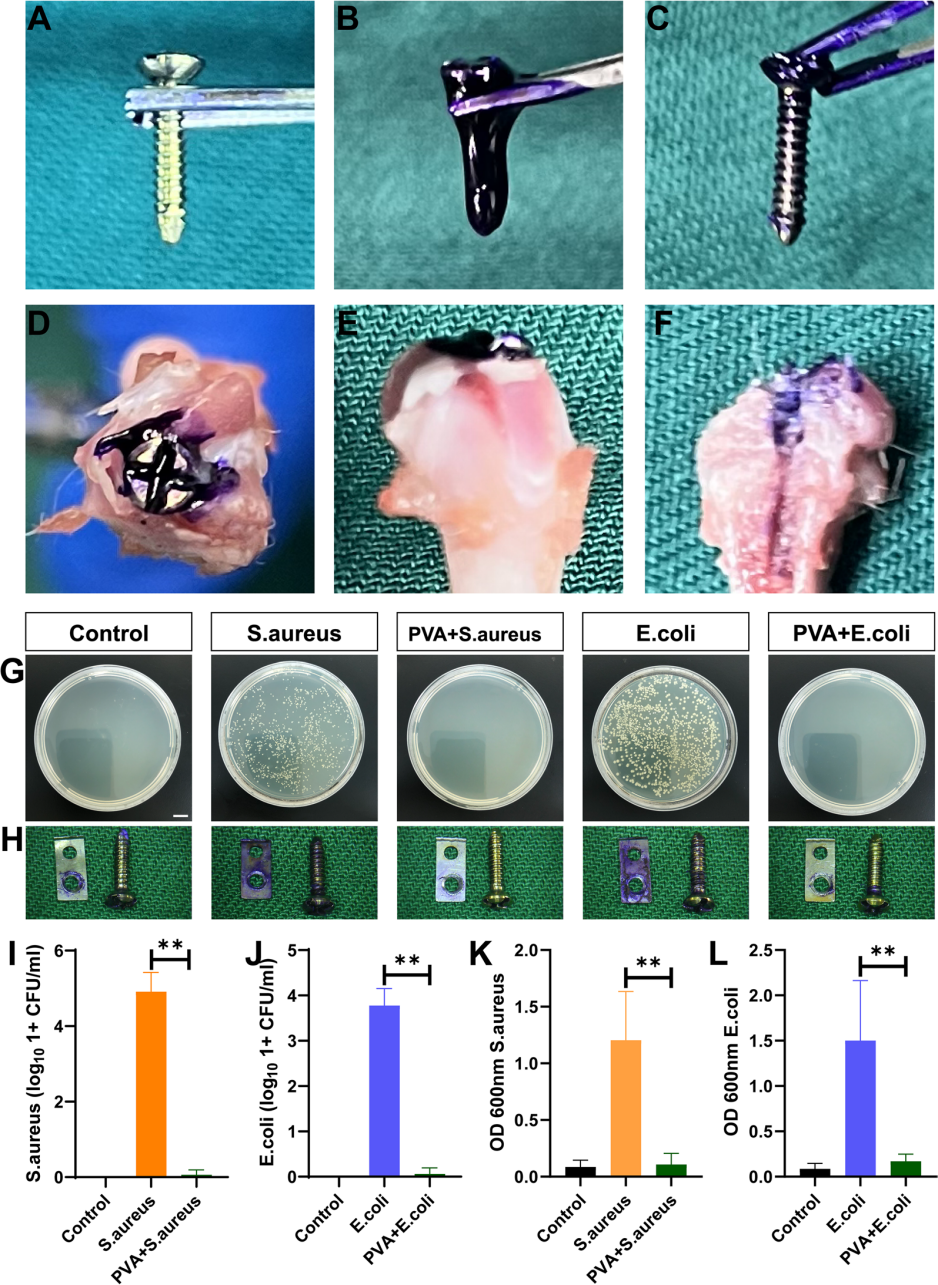
In Vivo Assessment of PVA Coating on Implants. **A** Screws before PVA coating. **B** Screws after PVA coating. **C** Screws after implantation and unscrewing, showing adherence of PVA coating. **D** and** E** General view after the screw is implanted into the distal femur. **F** Residue of PVA coating in the bone marrow cavity. **G** Plate coating 2 weeks after the operation (bar = 10 mm). **H** Crystal violet staining of the implant. **I** and** J** Quantitative results of colony formation around the implant. **K** and** L** Quantitative results of crystal violet staining on the implant Data are represented as means ± SD. Significant differences are indicated as **(*p* < 0.05)

To evaluate the adhesion of the PVA coating, we incorporated Alcian blue dye into the PVA coating (see Fig. 6A, B). The entire process, including the immediate coating of screws, implantation of free distal femurs in rats, and subsequent removal of screws, was meticulously documented. As depicted in Fig. 6C, the screw retained a blue stain after removal, confirming the adherence of the PVA coating to the screw surface. Figure 6F illustrates the presence of residual PVA coating within the bone marrow cavity, with the distribution of PVA diminishing as it extended deeper into the nail canal.

Additional file 1: Fig. S2 demonstrates a substantial reduction in the number of *S. aureus* colonies by 1.9 orders of magnitude and 3.4 orders of magnitude decrease in the number of *E. coli* colonies at the 2-h post-surgery time point. Crystal violet staining and quantitative analysis corroborate that PVA coating significantly mitigated biofilm formation on the implant surface, regardless of whether it was *S. aureus* or *E. coli*.

Additional file 1: Fig. S3 depicts colony formation around the implant and biofilm formation on the implant surface 24 h after surgery. The results reveal that in the PVA treatment group, the colony count of *S. aureus* increased from 10^3^ (at 2 h post-surgery) to 10^3.8^, while the colony count of *E. coli* increased from 10^2.6^ (at 2 h post-surgery) to 10^3.7^. However, when compared to the colony counts of *S. aureus* and *E. coli* at the same time point, they decreased by 3.8 and 3.7 orders of magnitude, respectively.

After 2 weeks post-operation (see Fig. 6G–L), infections around the implants in the PVA group had been effectively cleared, with only two colonies detected in one sample each from the PVA + *S. aureus* group and the PVA + *E. coli* group, respectively. In contrast, the colony counts in the *S. aureus* group and the *E. coli* group remained elevated at 10^4.9^ and 10^3.8^, respectively. The results of crystal violet staining were consistent with those observed at other time points, demonstrating a significant reduction in the number of biofilms on the implants in the PVA coating group.

### Assessing the protective effects of PVA coating on implants

**Fig. 7:**
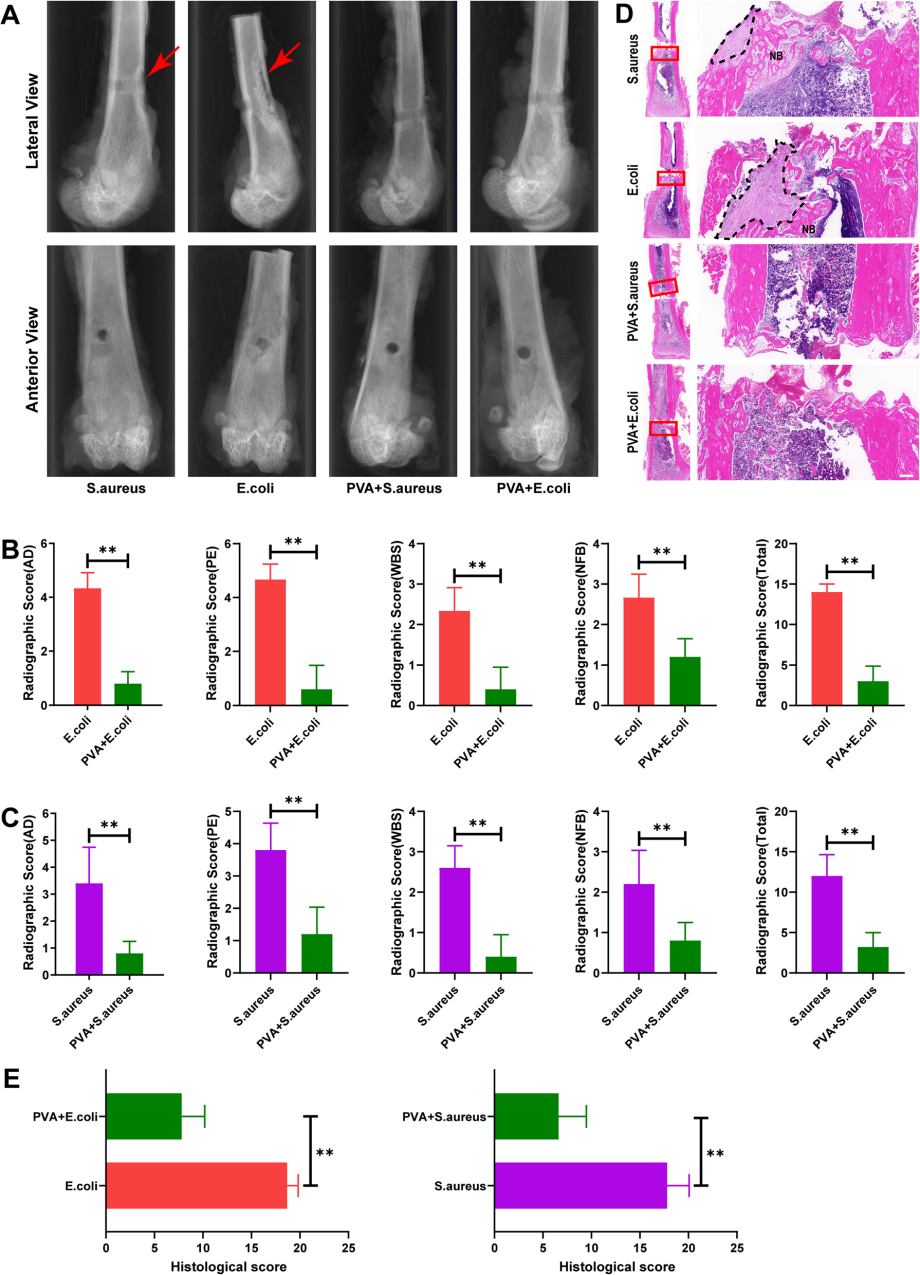
In Vivo Imaging and Histological Analysis at 2 weeks after surgery. **A** X-ray images of rat femurs, with the red arrow indicating the bone defect; **B** Imaging scores (including architectural deformation (AD), periosteal elevation (PE), widening of the bone shaft (WBS), new bone formation (NBF), and total score) of the control, *E. coli*, and PVA + *E. coli* groups; **C** Imaging scores (including architectural deformation (AD), periosteal elevation (PE), widening of the bone shaft (WBS), new bone formation (NBF), and total score) of the control *S. aureus* and PVA + *S. aureus* groups; **D** H-E staining of rat femurs, with the black dotted line representing the purulent lesion, and NB indicating new bone formation; bar = 200 μm. **E** Histological scores of the bacterial and PVA + bacterial groups. Data are presented as means ± SD. Significant differences are indicated as **(*p* < 0.05)

To further evaluate the protective impact of PVA coating on implants, rat femurs were selected for imaging and histological examination at the 2-week post-surgery mark. Figure 7 presents anteroposterior and lateral X-rays of the intact femur (Fig. 7A) and the comprehensive imaging scores (Fig. 7B, C), encompassing periosteal elevation (PE), architectural deformation (AD), widening of the bone shaft (WBS), new bone formation (NBF), and cumulative scores [48]. In the *S. aureus* and *E. coli* groups, femurs exhibited structural damage, characterized by severe periosteal reactions and prominent shadows of new bone formation. However, after PVA treatment, the bone destruction and periosteal reaction of the femur in the PVA + bacteria group decreased significantly. The scores of the PVA-coated group were notably lower than those of the bacteria-only group (Fig. 7B, C).

Histological evidence from each sample group further substantiated the efficacy of PVA. Hematoxylin and eosin (H&E) staining results (Fig. 7D) revealed the presence of infection foci in the femoral cortex of the bacteria-only group, with bacterial invasion leading to structural damage of the bone cortex. Attempted formation of artificial bone and fibrous tissue aimed to contain further destruction of the infected focus. However, rats coated with PVA displayed periosteal reactions and localized cortical bone damage in their femurs. Following Harrasser's methodology [49], we histologically scored the H&E staining results, considering (1) Granulocyte infiltration; (2) Sequestrum formation; (3) Mononuclear cell infiltration and bone marrow fibrosis; (4) Cortical bone enlargement; (5) Cortical bone erosion/destruction; and (6) Overall impression. As anticipated, the osteomyelitis score of rat femurs significantly decreased under the protection of PVA (Fig. 7E).

**Fig. 8:**
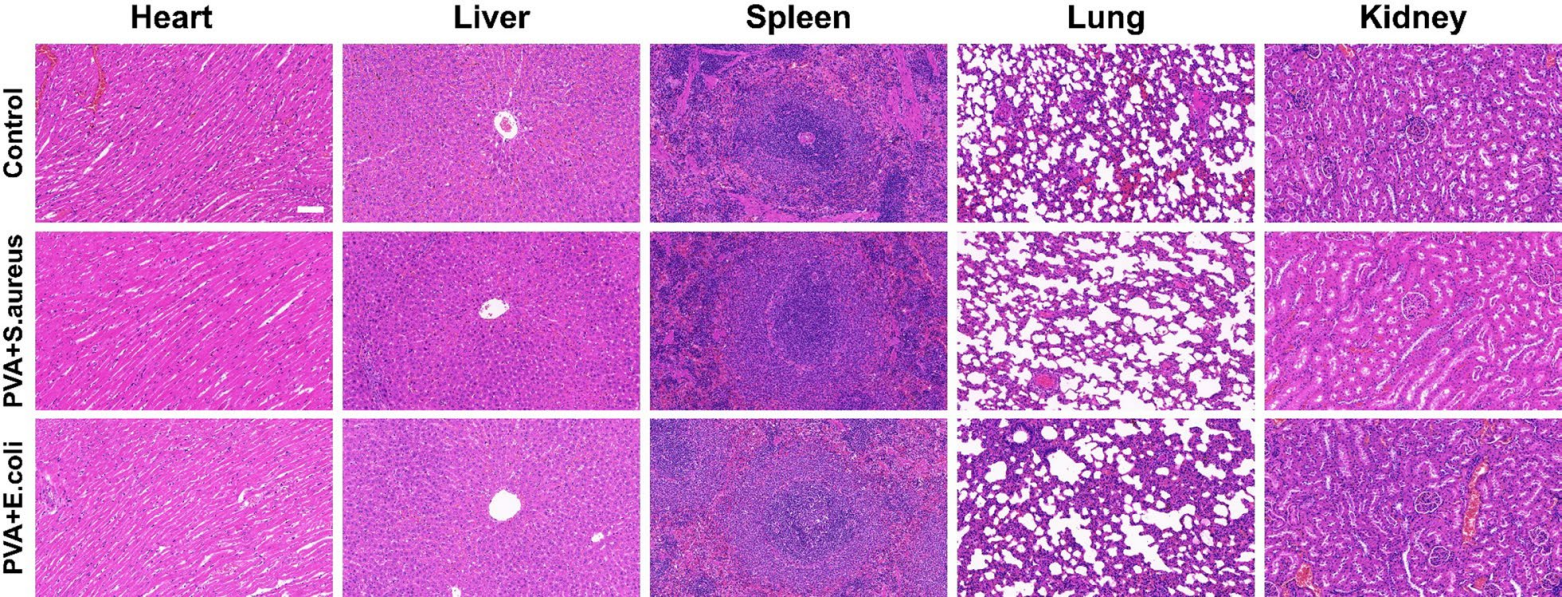
H-E staining of heart, liver, spleen, lung and kidney in rats; bar = 100 μm

Furthermore, compared to the control group, no evident abnormalities were observed in the liver, kidney, heart, spleen, and lung of the PVA-coated group, confirming the in vivo biosafety of PVA (Fig. 8).

## Discussion

Mechanisms of Bacterial Inhibition by PVA Solution:

In this study, we explored the potential of PVA solution, a widely utilized biomaterial, as a coating for implants aimed at preventing infections. We found that a 20% PVA solution exhibited enhanced effectiveness in restricting bacterial mobility and diffusion. Possible mechanisms are as follows.

In the PVA solution, the well-organized molecular structure of PVA and the strong hydrogen bonding between its molecules contribute to the creation of minute pore structures. These pores can impede bacterial diffusion, particularly for bacteria such as *E. coli* and *S. aureus*, which are significantly larger than the dimensions of these PVA pores. Consequently, bacteria may be constrained from easily penetrating the PVA layer.

The concentration of PVA in the solution is a critical factor. Higher PVA concentrations typically correspond to denser PVA layers. As PVA concentration increases, intermolecular interactions between PVA molecules in the solution, such as hydrogen bonding and van der Waals forces, become more pronounced. This results in molecular aggregation and clustering, densifying the solution's structure. As PVA concentration rises, spatial restrictions between polymer chains in the solution increase, hindering chain expansion. This restriction in expansion leads to higher polymer chain density and a more compact structure, subsequently elevating the elastic modulus and loss modulus of the solution. As seen in Fig. 1C–E, both the viscosity and elastic modulus of the solution increase, indicating enhanced intermolecular interactions and entanglement among PVA polymer chains, making it more challenging for bacteria to penetrate the PVA layer.

The thickness of the PVA layer also influences bacterial diffusion. A thicker PVA layer provides greater barrier properties, thereby more effectively preventing bacterial penetration through the PVA layer. As illustrated in Fig. 1A, a 20% PVA solution forms a thicker coating on the bottle surface.

The dissolution rate of PVA is another factor affecting its protective efficacy. As shown in Fig. 1B, the dissolution rate of PVA in PBS is inversely proportional to its concentration. The properties of the PVA solution and the characteristics of bacteria play significant roles in diffusion within the solution. Higher viscosity and concentration of PVA solution may hinder bacterial diffusion and slow down their movement within the solution. It's important to note that higher-concentration PVA solutions tend to be more viscous and have slower dilution rates. Conversely, lower-concentration PVA solutions are easier to dilute. For orthopedic surgeries that typically require extended durations, higher-concentration PVA solutions may be employed. As the PVA solution is diluted, its viscosity decreases, facilitating drainage from the wound site through drainage tubes or aspirators.

Li et al. demonstrated that the combination of PVA solution and antibiotics effectively prevented infections caused by contaminated implants in vivo [50]. Similarly, another study reported the prevention of biofilm formation by a commercial adhesive coating combined with antibiotics, although the underlying mechanism remains unclear [51–54]. In addition, Johnson et al. developed a hydrogel loaded with lysostaphin to prevent implant infection and promote bone regeneration, but it is only effective against *staphylococci* [55]. As a result, in our in vivo experiments, we observed a protective effect of PVA as early as 2 h after the operation, and after 2 weeks, approximately 80% of the rats achieved complete bacterial clearance around the implants. We propose that PVA obstructs bacterial attachment and biofilm formation, thereby depriving bacteria of the protective environment provided by the biofilm.

In summary, our study provides compelling evidence for the antibacterial properties of PVA and its potential application in preventing implant-related infections. Further research is warranted to delve into the underlying mechanisms and optimize the formulation of PVA-based antibacterial materials for clinical use.

The safety of PVA:

The safety of implant coatings is a paramount consideration. Regarding PVA, its biosafety has been rigorously investigated and confirmed. Several key findings support the safety of PVA:

Accumulation and Elimination: PVA does not accumulate in the body, whether administered orally or intravenously. It is efficiently excreted through the kidneys in urine [56, 57]. The half-life of PVA depends on its molecular weight, and the PVA solution used in this study has a relatively short half-life of less than 50 min [56].

Genetic toxicity: PVA has been found to be non-genotoxic in various studies [58, 59]. This indicates that it does not cause damage to the genetic material of cells. Due to its established safety profile, PVA has been approved by the FDA for clinical use in humans [29]. In line with these findings, our in vivo experiments demonstrated that the topical application of PVA solution did not induce toxicity in organs such as the liver, kidneys, and myocardium of rats.

In the development of implant coatings, another important consideration is their effect on osseointegration [60]. It is crucial that the coating does not negatively impact the microenvironment necessary for successful osseointegration. In our study, we evaluated the toxicity of PVA solution on chondrocytes, osteoblast-like cells (MC3T3-E1), and macrophages (RAW267.4). Except for macrophages, the survival rate of cells exposed to PVA was close to 100%. It is worth noting that RAW264.7 cells have a short proliferation cycle and high nutrient demands. The decreased survival rate of RAW264.7 cells in the presence of PVA may be attributed to the potential blocking of oxygen delivery by PVA [61]. Additionally, PVA did not promote or inhibit the osteogenic differentiation of MC3T3-E1 cells, as observed in Fig. 5. Taken together, the extensive evidence on the biosafety of PVA, as well as our experimental results, support the suitability of PVA as a safe implant coating material with no adverse effects on cellular viability and osseointegration.

Indeed, there are several limitations to our study that should be acknowledged. Limited bactericidal activity: Our study showed that PVA can inhibit the proliferation of *S. aureus* and *E. coli* and prevent biofilm formation. However, the exact mechanism by which PVA inhibits bacterial growth and whether it can effectively clear mature biofilms require further investigation. Understanding these mechanisms would provide valuable insights into the antibacterial properties of PVA. Scope of antibacterial activity: Although our results indicate that PVA is effective against *S. aureus* and *E. coli*, it is important to evaluate its efficacy against a broader range of bacterial strains. Testing PVA against other clinically relevant bacteria would help refine its antibacterial spectrum and assess its potential as a broad-spectrum antimicrobial agent. Validation in different animal models: While our study utilized a rat model with distal femur implantation, it is necessary to validate the findings in other animal models, such as those involving prosthetic implantation or spinal internal fixation systems. Different anatomical locations and implant types may present distinct challenges in terms of infection prevention and treatment, and evaluating the efficacy of PVA coatings in these models would provide a more comprehensive understanding of its potential applications.

## Conclusion

In summary, our study underscores the considerable potential of PVA solution as a versatile biomaterial in the realm of implant-associated infection prevention and treatment. PVA emerges as a formidable candidate, manifesting substantial antibacterial prowess through its adept inhibition of biofilm formation and proliferation of both *S. aureus* and *E. coli*. It is worth highlighting that PVA solution, in addition to its robust antibacterial properties, upholds a commendable degree of biocompatibility, devoid of any detrimental impact on the vitality or osteogenic differentiation of osteoblast-related cells.

In our comprehensive in vivo assessments, PVA solution proved to be a formidable deterrent against bacterial colonization and biofilm formation on implant surfaces, exhibiting efficacy across different time intervals. These compelling findings collectively propose that PVA solution stands as an attractive prospect, offering a cost-effective and readily accessible biomaterial for clinical deployment in the relentless battle against implant-associated infections.

### Supplementary Information

**Additional file 1: Fig. S1.** The process of coating titanium plate with 5% PVA. **Fig. S2**. In vivo assessment of PVA coating on implants 2 h after operation. **A** Plate coating 2 weeks after the operation, bar = 10 mm. **B** Crystal violet staining of the implant. **C** and **D** Quantitative results of colony formation around the implant. **E** and **F** Quantitative results of crystal violet staining on the implant Data are represented as means ± SD. Significant differences are indicated as **(*p* < 0.05). **Fig. S3.** In vivo assessment of PVA coating on implants 24 h after operation. **A** Plate coating 2 weeks after the operation, bar = 10 mm. **B** Crystal violet staining of the implant. **C** and **D** Quantitative results of colony formation around the implant. **E** and **F** Quantitative results of crystal violet staining on the implant Data are represented as means ± SD. Significant differences are indicated as **(*p* < 0.05).

## Data Availability

Not applicable.
