# Supplementary material for: A biodegradable PVA coating constructed on the surface of the implant for preventing bacterial colonization and biofilm formation
Source: J Orthop Surg Res. 2024 Mar 8;19:175. doi: 10.1186/s13018-024-04662-7 (PMC10921624; doi:10.1186/s13018-024-04662-7)
Supplement: Supplementary file 1 — Additional file 1: Fig. S1. The process of coating titanium plate with 5% PVA. Fig. S2. In vivo assessment of PVA coating on implants 2 h after operation. A Plate coating 2 weeks after the operation, bar = 10 mm. B Crystal violet staining of the implant. C and D Quantitative results of colony formation around the implant. E and F Quantitative results of crystal violet staining on the implant Data are represented as means ± SD. Significant differences are indicated as **(p < 0.05). Fig. S3. In vivo assessment of PVA coating on implants 24 h after operation. A Plate coating 2 weeks after the operation, bar = 10 mm. B Crystal violet staining of the implant. C and D Quantitative results of colony formation around the implant. E and F Quantitative results of crystal violet staining on the implant Data are represented as means ± SD. Significant differences are indicated as **(p < 0.05). [file 13018_2024_4662_MOESM1_ESM.docx]

**A biodegradable PVA coating constructed on the surface of the implant for preventing bacterial colonization and biofilm formation**

Zhonghua Lei^1,2#^, Haifeng Liang^1,3#^, Wei Sun^1^, Yan Chen^4^, Zhi Huang^5^*, and Bo Yu^1^*,

1. Orthopedic and traumatology department, Zhujiang Hospital, Southern Medical University, Guangzhou, China 510282;

2. Department of Orthopedics, The Sixth Peoples Hospital of Huizhou, Huizhou, China 516211;

3. Department of Orthopedics, The Third Affiliated Hospital of Guangzhou Medical University, Guangzhou, China 510150;

4. Ultrasound Medical Center, Zhujiang Hospital, Southern Medical University, Guangzhou, China 510282;

5. Institute of Biomedical Engineering, School of Basic Medical Sciences, Central South University, Changsha, China 410083;

^#^The first two authors contributed equally to this manuscript.

* Correspondence: Zhi Huang, [biomaterials@csu.edu.cn](mailto:biomaterials@csu.edu.cn); Tel.: +86-13117512783. Bo Yu, gzyubo@smu.edu.cn; Tel.: +86-13828434375.


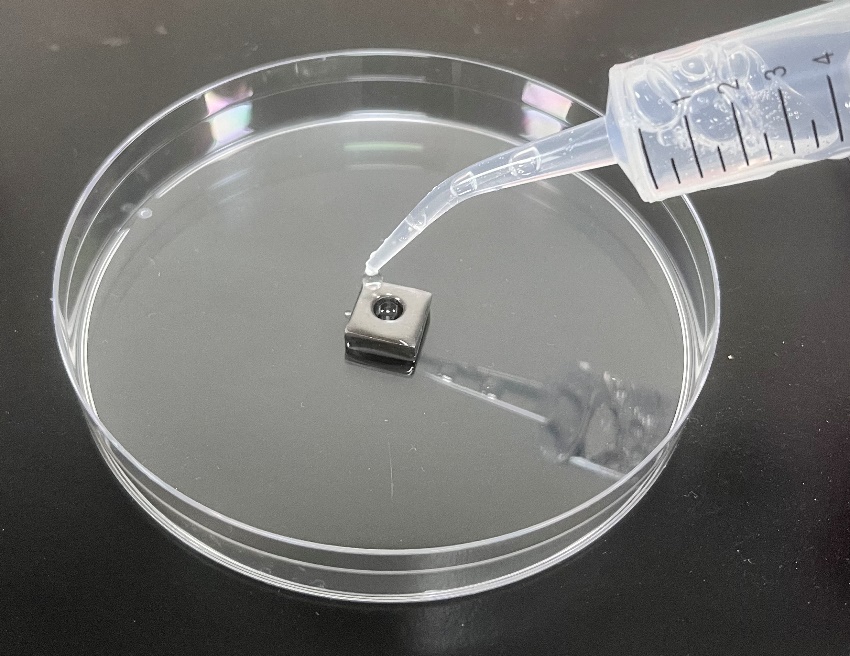


Figure S1 The process of coating titanium plate with 5% PVA

Figure S2 *In Vivo* Assessment of PVA Coating on Implants 2h after operation. **A** Plate coating two weeks after the operation, bar=10mm. **B** Crystal violet staining of the implant. **C** and **D** Quantitative results of colony formation around the implant. **E** and **F** Quantitative results of crystal violet staining on the implant Data are represented as means ± SD. Significant differences are indicated as ** (p < 0.05).

Figure S3 *In Vivo* Assessment of PVA Coating on Implants 24h after operation. **A** Plate coating two weeks after the operation, bar=10mm. **B** Crystal violet staining of the implant. **C** and **D** Quantitative results of colony formation around the implant. **E** and **F** Quantitative results of crystal violet staining on the implant Data are represented as means ± SD. Significant differences are indicated as ** (p < 0.05).
